# Supplementary figures and images for: New information on the early Permian lanthanosuchoid Feeserpeton oklahomensis based on computed tomography
Source: PeerJ. 2019 Oct 31;7:e7753. doi: 10.7717/peerj.7753 (PMC6825742; doi:10.7717/peerj.7753)

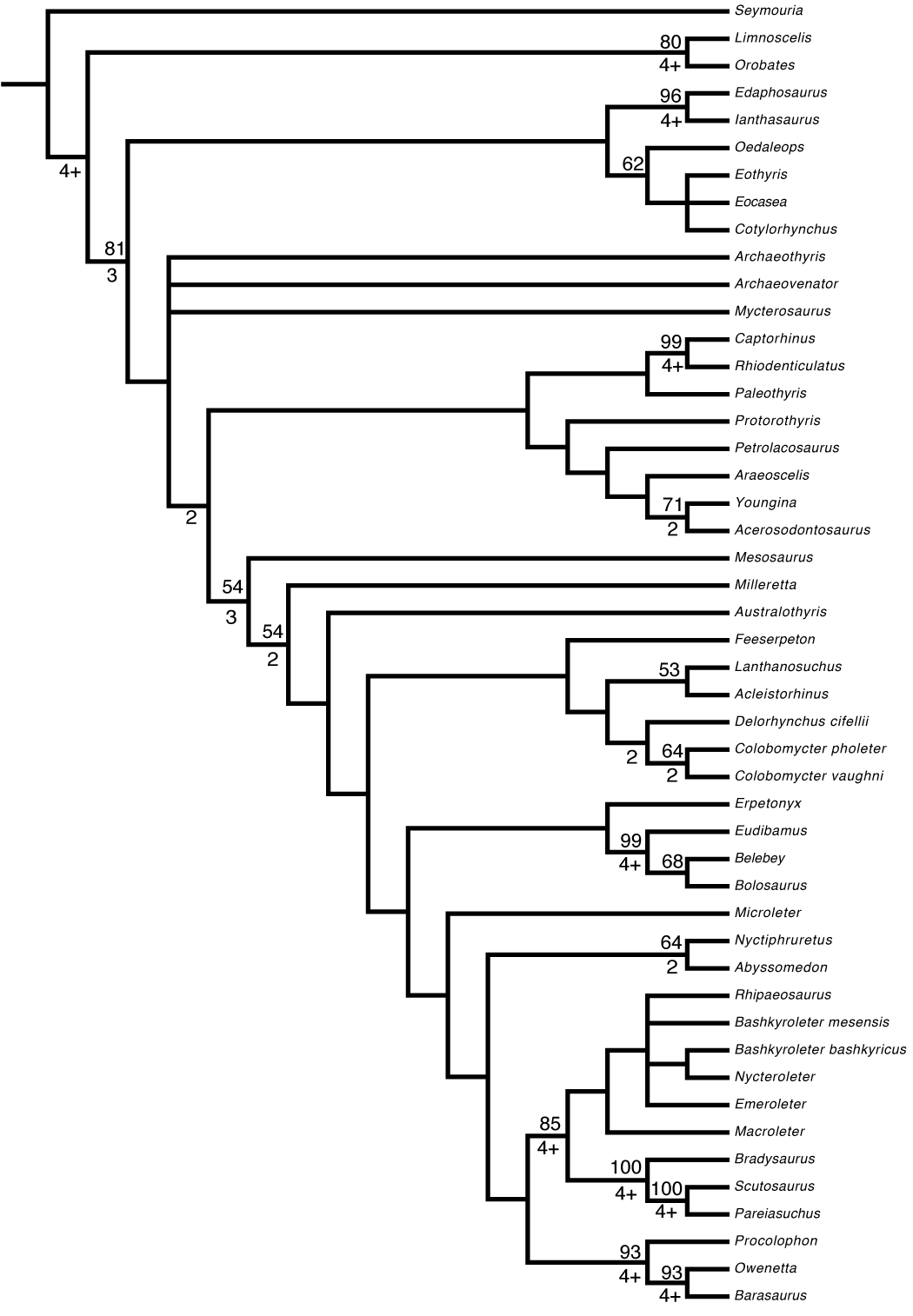

Supplement: Supplemental Information 1 — Tree length = 674, consistency index = 0.298, rescaled consistency index = 0.193, retention index = 0.646. Bootstrap support values are found above nodes, if no value is indicated it was less than 50%. Bremer support values are found below nodes, if no value is indicated the clade collapsed with the addition of one extra step. [file peerj-07-7753-s001.png]
