# Supplementary material for: New information on the early Permian lanthanosuchoid Feeserpeton oklahomensis based on computed tomography
Source: PeerJ. 2019 Oct 31;7:e7753. doi: 10.7717/peerj.7753 (PMC6825742; doi:10.7717/peerj.7753)
Supplement: Supplemental Information 3 [file peerj-07-7753-s003.docx]

APPENDIX 1

List of characters used in the phylogenetic analysis.

(1) Narial shelf: absent (0); present (1). (Modified from Laurin & Reisz, 1995 #1)

(2) If narial shelf is present, it is: found on the nasal (0); found on the nasal and maxilla (1). (Modified from Laurin & Reisz, 1995 #1)

(3) Prefrontal-postfrontal contact in dorsal view: present (0); absent (1). (Modified from Laurin & Reisz, 1995 #2)

(4) If prefrontal-postfrontal contact is absent, the frontal contribution to the orbital margin in dorsal view is: narrow (0); broad (1). (Modified from Laurin & Reisz, 1995 #2) When the frontal contribution is narrow, the frontal occupies less than 1/3 of the dorsal orbital margin.

(5) Frontal lateral lappet: absent (0); present (1). (Modified from deBraga & Reisz, 1996 #7)

(6) Pineal foramen position: in the middle of the body of the parietal (0); displaced posteriorly (1); displaced anteriorly (2). (Modified from deBraga & Rieppel, 1997 #49)

(7) Postparietal: present (0); absent (1). (Modified from Laurin & Reisz, 1995 #4)

(8) If postparietal is present, it is: paired (0); median (1). (Modified from Laurin & Reisz, 1995 #4)

(9) If postparietal is present, its size is: large (0); small (1). (Modified from Laurin & Reisz, 1995 #4). Postparietal (either both paired elements together or a single median one) is large when it is at least the width of one of the parietals (at their widest point).

(10) If postparietal is present, it is positioned such that it is: dorsally exposed, integrated into skull table (0); occipital (1). (Laurin & Reisz, 1995 #5)

(11) Prefrontal-palatal contact: absent (0); present (1). (Modified from Laurin & Reisz 1995 #6)

(12) If prefrontal-palatal contact is present, it is: narrow and acuminate (0); strong, with sutural base (1). (Modified from Laurin & Reisz, 1995 #6)

(13) Prefrontal medial flange: narrow (0); wide (1). (Modified from Laurin & Reisz 1995 #7) When narrow, the mediolateral length of the prefrontal medial flange is equal to or less than 1/5 the height of the orbit.

(14) Bulbous medial process of prefrontal: absent (0); present (1). (Laurin & Reisz, 1995 #8)

(15) Lacrimal narial contact: present (0); absent (1). (Laurin & Reisz, 1995 #9)

(16) Foramen orbitonasale: absent (0); present (1). (Modified from Laurin & Reisz, 1995 #10)

(17) If foramen orbitonasale is present, it is: represented by a medial indentation on the lacrimal and a dorsal indentation on the palatine (0); enclosed between prefrontal, lacrimal and palatine (1). (Modified from Laurin & Reisz, 1995 #10)

(18) Jugal anterior process: does not extend to anterior orbital rim (0); extends at least to level of anterior orbital rim (1). (Laurin & Reisz, 1995 #11)

(19) Suborbital ramus of jugal: dorsoventrally broad (0); slender or reduced with no dorsoventral extension (1). (MacDougall & Reisz, 2014) When the suborbital ramus of the jugal is slender, its dorsoventral height is less than that of the dorsoventral height of the suborbital portion of the maxilla.

(20) Posterior extension of jugal that contributes to temporal region of skull roof: present (0); absent (1). (MacDougall & Reisz, 2014)

(21) Postorbital posterior process shape in lateral view: slender, anteroposterior length is at least twice its dorsoventral width (0); increased width, length is less than twice its width (1). (Modified from deBraga & Reisz, 1996 #14)

(22) Postorbital-supratemporal contact: present (0); absent (1). (Modified from Laurin & Reisz, 1995 #12)

(23) Posterolateral corner of skull roof; formed by tabular (0); formed mostly by supratemporal (1); formed by parietal and small supratemporal or parietal alone (2). (Laurin & Reisz, 1995 #15)

(24) Tabular: present (0); absent (1). (Modified from Laurin & Reisz, 1995 #17)

(25) If tabular is present, it is: part of skull table (0); largely occipital (1). (Modified from Laurin and Reisz, 1995 #17)

(26) Supratemporal: present (0); absent (1). (Modified from Laurin & Reisz, 1995 #18)

(27) If supratemporal is present, it is: large (0); small (1). (Modified from Laurin & Reisz, 1995 #18) When small the mediolateral width of the element is equal to or less than 1/10 of the midline skull length.

(28) Premaxillary dorsalf process: broad, narial opening faces predominantly laterally (0); narrow, narial opening faces anteriorly (1). (deBraga & Reisz, 1996 #1)

(29) Anterodorsal process of the maxilla: absent (0); present (1). (Modified from Laurin & Reisz, 1995 #19)

(30) Anterior lateral maxillary foramen: equal in size to other maxillary foramina (0); much larger than other foramina (1). (Modified from Laurin & Reisz, 1995 #20)

(31) Maxilla and quadratojugal: in contact (0); separated (1). (Laurin & Reisz, 1995 #22)

(32) Suture between jugal and maxilla: straight, jugal thins out smoothly towards anterior direction (0); ‘stepped’, anterior most tip of jugal very narrow but expands broadly posteriorly along with a dramatic thinning of the posterior process of the maxilla (1). (Müller & Tsuji, 2007 #133)

(33) Contact between maxilla and prefrontal: absent (0); present (1). (Müller & Tsuji, 2007 #136)

(34) Contribution of maxilla to external naris: maxilla is either excluded from naris or forms only its ventral/posterior edge (0); maxilla extends also to the posterodorsal margin of naris (1). (Müller & Tsuji, 2007 #137)

(35) Presence of size related heterodonty on the anterior 2/3 of the maxilla: present (0); absent (1). (MacDougall & Reisz, 2014). Size related heterodonty as used here refers to the presence of significantly larger teeth on the anterior 2/3 of the maxilla. Significantly larger as used here refers to teeth that have a base width that is at least 50% larger than the other teeth.

(36) Presence of size related heterodonty on the anterior 2/3 of the dentary: present (0); absent (1). (MacDougall & Reisz, 2014) Size related heterodonty as used here refers to the presence of significantly larger teeth on the anterior 2/3 of the dentary. Significantly larger as used here refers to teeth that have a base width that is at least 50% larger than the other teeth.

(37) If size related heterodonty is present on the maxilla, it is represented by: one tooth (0); two or more teeth (1). (MacDougall & Reisz, 2014)

(38) Squamosal and post-temporal fenestra: separated (0); in contact (1). (Laurin and Reisz, 1995 #26)

(39) Quadratojugal shape: does not reach beyond the level of the ventral orbital margin (0); extends dorsally beyond the level of ventral orbital margin (1). (Modified from Laurin & Reisz, 1995 #28)

(40) Quadratojugal anterior extent: reaches posterior border of orbit (0); does not reach level of posterior border of orbit (1). (Modified from Laurin & Reisz, 1995 #23)

(41) Quadratojugal ornamentation: confluent with the cheek and not ornate in any manner (0); ornamented, dermal protuberances project from its surface (1). (deBraga & Rieppel, 1997 #43)

(42) Upper temporal fenestra: absent (0); present (1). The upper temporal fenestra is formed by contributions from the postorbital, the parietal, the squamosal. (Laurin & Reisz, 1995 #29)

(43) Lower lateral temporal opening (fenestra or emargination): absent (0); present (1). (Modified from Laurin & Reisz, 1995 #30)

(44) If a lower temporal opening is present, it is: bounded ventrally, forming a fenestra (0); unbounded ventrally, forming an emargination (1). (Modified from Laurin & Reisz, 1995 #30)

(45) Postorbital contribution to lower lateral temporal opening (fenestra or emargination): present (0); absent (1). (Modified from deBraga & Reisz, 1996 #20)

(46) Quadratojugal contribution to lower lateral temporal opening (fenestra or emargination): absent (0), present (1). (Modified from Laurin & Reisz, 1995 #16)

(47) Region of skull posterior to the orbit, anteroposterior length: equals anteroposterior extension of orbit (0); shorter than anteroposterior extension of orbit (1); longer than anteroposterior extension of orbit (2). (Modified from Laurin & Reisz, 1995 #32)

(48) Ventral margin of skull region posterior to orbit: expanded below ventral extent of maxilla (0); rectilinear (1); emarginated (2). (Modified from Laurin & Reisz, 1995 #33)

(49) Lateral surface of quadrate: covered by squamosal and quadratojugal (0); not covered (1). (Modified from Laurin & Reisz, 1995 #34)

(50) Quadrate anterior process: extends anteriorly for at least 50% the length of the quadrate ramus (0); extends anteriorly for less than 50% the length of the quadrate ramus (1). (Modified from Laurin & Reisz, 1995 #35)

(51) Quadrate condyle articular surfaces: strongly convex, anteroposteriorly longer than they are wide (0); nearly flat, anteroposteriorly shorter than they are wide (1). (Modified from Laurin & Reisz, 1995 #65)

(52) Jaw articulation position: posterior to occiput (0); even with occiput (1); anterior to occiput (2). (Laurin & Reisz, 1995 #36)

(53) Posterior extension of orbit: absent (0); present (1). (Laurin & Reisz, 1995 #37)

(54) Dermal sculpturing: absent (0); present (1). (Modified from Laurin & Reisz, 1995 #38)

(55) If dermal sculpturing is present, it is in the form of: tuberosities (0); round pits (1); honeycomb pattern of ridges and pits (2). (Modified from Laurin & Reisz, 1995 #38)

(56) Sculpturing involving circumorbital bumps: no distinctive ornamentation (0); circumorbital tubercles (1). (Tsuji, 2006 #45)

(57) Dorsal dermal ossifications: absent (0); present (1). (Laurin & Reisz, 1995 #124)

(58) Posterior margin of skull roof: roughly straight (0); with a single, median embayment (1); embayed bilaterally (2). (Modesto, 1999 #125)

(59) Temporal notch: present (0); absent (1). (Müller & Tsuji, 2007 #134)

(60) Temporal depression associated with posterolateral excavation: restricted to the posterior half of the cheek (0); closely approaches the orbital margin (1). (Müller & Tsuji, 2007 #135)

(61) Interpterygoid vacuity anterior extent: reaches beyond posterior border of palatine (0); reaches level of palatine or less (1). (Modified from Reisz et al., 2007 #127)

(62) Anterior shape of interpterygoid vacuity: acuminate (0); rounded (1).

(63) Choana: parallel to maxilla; palatine forms its posterior edge only (0); curved posteromedially; palatine forms its posterior and part of its lateral edge (1). (Laurin & Reisz, 1995 #40)

(64) Alar flange of the vomer (thin anterolateral flange of vomer): absent (0); present (1). (Tsuji, 2006 #50)

(65) Arcuate flange of pterygoid: present (0); absent (1). (Laurin & Reisz, 1995 #42)

(66) Cranio-quadrate space: small, quadrate ramus of pterygoid and paraoccipital process of opisthotic converge posterolaterally (0); large, quadrate ramus of pterygoid and paraoccipital process of opisthotic are parallel to each other (1). (Laurin & Reisz, 1995 #43)

(67) Pterygoid anterior extent: reaches level of posterior end of choana (0); posterior to choana (1). (Laurin and Reisz, 1995 #44)

(68) Transverse flange of the pterygoid: large, approaches cheek, a noticeable lateral projection (0); small, does not approach cheek (1). (Modified from Lee, 1997 #19)

(69) Transverse flange of pterygoid orientation: directed posterolaterally or transversely (0); directed anterolaterally (1); directed anteriorly (2). (Modified from Laurin & Reisz, 1995 #45)

(70) Transverse flange of pterygoid dentition: present (0); absent (1). (Modified from Laurin & Reisz, 1995 #46)

(71) If dentition is present on the transverse flange of the pterygoid, it is a: shagreen of very small teeth, no ventral ridge (0); single row of large teeth, no ventral ridge (1); single row of large teeth, with a shagreen of very small teeth, no ventral ridge (2). (Modified from Laurin & Reisz, 1995 #46)

(72) Quadrate flange of pterygoid dentition: absent (0); present (1).

(73) Quadrate ramus of pterygoid: merges smoothly into transverse flange without distinctive excavation (0); deep excavation on posterolateral surface (1). (deBraga & Reisz, 1996 #29)

(74) Quadrate ramus of pterygoid relation to trasverse flange: not continuous with transverse flange (0); continuous with transverse flange, forming a ridge (1).

(75) Lateral pocket on the pterygoid found between quadrate ramus and transverse flange: absent (0); present (1).

(76) Ectopterygoid: present (0); absent (1). (MacDougall & Reisz, 2014)

(77) If ectopterygoid is present, ectopterygoid dentition is: present (0); absent (1). (Modified from Laurin & Reisz, 1995 #48)

(78) If ectopterygoid is present, its relationship to transverse flange: distal to transverse flange, does not contribute to lateral portion of flange (0); makes contact with lateral portion of transverse flange (1). (Modified from deBraga & Reisz, 1996 #33)

(79) Suborbital opening on the palate: absent (0); present (1). (Modified from Laurin & Reisz, 1995 #49) Refers to the suborbital foramen or fenestra.

(80) Basicranial articulation: kinetic/synovial (0); sutured and/or immobile (1). (Modified from Lee, 1997 #2)

(81) Length of basicranial articulation: restricted to anterolateral margin of the parasphenoid (0); extends over much of length of main body of parasphenoid (1). (deBraga & Reisz, 1996 #36)

(82) Parasphenoid pocket for cervical musculature (one or two): present (0); absent (1). (Laurin & Reisz, 1995 #50)

(83) If a parasphenoid pocket for cervical musculature is present it is represented by: a single median pocket (0); two pockets (1).

(84) Parasphenoid wings; present, parasphenoid broader posteriorly than long (0); absent, parasphenoid narrower posteriorly than long (1). (Modified from Laurin & Reisz, 1995 #51)

(85) Cultriform process: present (0); absent (1). (Modified from Laurin & Reisz, 1995 #52)

(86) If cultriform process is present, it is: longer than the body of the parasphenoid (0); shorter than the body of the parasphenoid (1). (Modified from Laurin & Reisz, 1995 #52)

(87) Parasphenoid teeth: absent (0); present (1). (Modified from Laurin & Reisz, 1995 #53)

(88) If parasphenoid teeth are present, they are found: in rows (0); as a shagreen (1). (Modified from Laurin & Reisz, 1995 #53)

(89) Supraoccipital: plate-like, with no sagittal crest (0); constricted at midline, forming sagittal crest (1); plate-like, with a saggital crest (2). (Modified from deBraga & Rieppel, 1997 #56)

(90) Paroccipital process: vertically broad (0); anteroposteriorly expanded (1); narrow (2); tubular, composed of opisthotic (3). (Laurin & Reisz, 1995 #56)

(91) Paroccipital process orientation: directed primarily laterally (0); oriented obliquely, at an angle of at least 45 degrees from the horizontal plane of the skull (1). (deBraga & Reisz, 1996 #44)

(92) Sutural contact between paroccipital process and dermatocranium: absent (0); present (1). (Modified Laurin & Reisz, 1995 #57)

(93) Otic trough in ventral flange of opisthotic: absent (0); present (1). (Laurin & Reisz 1995 #58)

(94) Medial wall of inner ear (made of prootic): unossified (0); ossified with acoustic nerve foramina (1). (Laurin & Reisz, 1995 #59)

(95) Post-temporal fenestra: absent (0); present (1). (Modified from deBraga & Rieppel, 1997 #59)

(96) If post-temporal fenestra is present, it is: small, diameter less than the diameter of foramen magnum (0); large, diameter at least equal to foramen magnum (1). (Modified from deBraga & Rieppel, 1997 #59)

(97) Osseous contact between basioccipital and basisphenoid: present (0); absent (1). (Lee, 1993 #A3, scored as per Laurin & Reisz, 1995 #61)

(98) Occipital condyle shape: transversely broad (0); reniform to circular (1). (Laurin & Reisz, 1995 #62)

(99) Ventral exposure of basioccipital: contributes extensively to ventral surface of the braincase (0); restricted to condylar region (1). (deBraga & Reisz, 1996 #37)

(100) Ventral braincase tubera: absent (0); present (1). (Modified from Laurin & Reisz, 1995 #63, and deBraga and Rieppel 1997 #65)

(101) If ventral braincase tubera are present, they are: restricted to basioccipital (0); very large and restricted to basisphenoid (1). (Modified from Laurin and Reisz, 1995 #63, and deBraga & Rieppel, 1997 #65)

(102) Lateral flange of exoccipital: absent (0); present (1). (Laurin & Reisz, 1995 #64)

(103) Stapes: robust, greatest depth exceeding one-third of total length (0); slender, length at least four times depth (1). (Modified from deBraga & Rieppel, 1997 #45)

(104) Stapedial dorsal process: ossified (0); unossified (1). (Laurin and Reisz, 1995 #67)

(105) Morphology of marginal dentition: single cusp (0); two to seven cusps (1); more than seven cusps (2). (Modified from Lee 1997, #59)

(106) Foramen intermandibularis: an anterior symphysial foramen (0); an anterior symphysial foramen and a posterior foramen (1). (Modified from Laurin & Reisz, 1995 #69)

(107) If there are two intermandibular foramina the posterior foramen is located: anterior to coronoid process (0); posterior to or at level of coronoid process (1). (Modified from Laurin & Reisz, 1995 #69)

(108) Meckelian fossa orientation: faces mediodorsally (0); faces dorsally (1). (Modified from Laurin & Reisz, 1995 #70)

(109) Meckelian fossa anteroposterior length: long, occupies at least 25% of lower jaw length (0); short, occupies less than 25% of lower jaw length (1). (Modified from Laurin & Reisz, 1995 #71)

(110) Surangular length: extends beyond coronoid eminence (0); does not extend beyond coronoid eminence (1). (Laurin & Reisz, 1995 #72)

(111) Accessory lateral shelf on surangular anterior to articular region: absent (0); present (1). (Laurin & Reisz, 1995 #73)

(112) Two laterally located foramina on the anteroposterior midline of the surangular: absent (0); present (1).

(113) Coronoid number: two or three (0); one (1). (Laurin & Reisz, 1995 #74)

(114) Prearticular extends: beyond the coronoid eminence (0); does not extend beyond coronoid eminence (1). (Modified from Laurin & Reisz, 1995 #75)

(115) Retroarticular process: present (0); absent (1). (Modified from Laurin & Reisz, 1995 #76)

(116) If present, the retroarticular process is: small and narrow (0); transversely broad, dorsally concave (1). (Modified from Laurin & Reisz, 1995 #76)

(117) If present, the retroarticular process is composed of: articular body (0); three or more elements (articular, prearticular, angular and surangular) (1). (Modified from Laurin & Reisz, 1995 #77)

(118) Lateral shelf on articular region: absent (0); present (1). (Modified from Laurin & Reisz, 1995 #78)

(119) Coronoid process: low (0), high (1). (Modified from Laurin & Reisz, 1995 #79)

(120) If coronoid process is high, it is: composed of coronoid only (0); composed of dentary and coronoid (1). (Modified from Laurin & Reisz, 1995 #79)

(121) Splenial: contributes to mandibular symphysis (0); excluded from mandibular symphysis (1). (Laurin & Reisz, 1995 #80)

(122) Presacral vertebral count: more than twenty (0); twenty or less (1). (Laurin & Reisz, 1995 #81)

(123) Axial centrum orientation: in plane of axial skeleton (0); sloping anterodorsally (1). (Laurin & Reisz, 1995 #82)

(124) Atlantal neural arch: possesses epipophysis (0); lacks epipophysis (1). (Lee 1995, scored as per Modesto, 1999 #126)

(125) Axial intercentrum: with rounded anteroventral edge (0); with strong anterior process (1). (Laurin & Reisz, 1995 #84)

(126) Atlantal pleurocentrum and axial intercentrum: separate elements (0); attached or fused (1). (Laurin & Reisz, 1995 #85)

(127) Trunk neural arches: swollen (0); narrow (1). (Modified from Laurin & Reisz, 1995 #86)

(128) Ventral surface of anterior pleurocentra: ventral surface of vertebral centra uniform (0); ventral surface of vertebral centra bearing an excavation on either side of the midline, coupled with a flattened median crest between them (1). (Modified from Laurin & Reisz, 1995 #87)

(129) Number of sacral vertebrae: one (0); two (1); three or more (2). (Laurin & Reisz, 1995 #88)

(130) Sacral rib distal overlap: broad with narrow gap between ribs (0); small or absent with wide gap between ribs (1). (Laurin & Reisz, 1995 #89)

(131) Transverse process or ribs: present only on a few anterior caudals (0); present on at least thirteen caudals (1). (Laurin & Reisz, 1995 #90)

(132) Anterior caudal rib size: elongate and extend posteriorly to the end of the next vertebra (0); curve posteriorly but do not extend to the end of the next vertebrae (1); straight, with no posterior curvature (2).

(133) Caudal hemal arches: wedged between centra (0); attached to anterior centrum (1). (Laurin & Reisz, 1995 #91)

(134) Interclavicle: diamond-shaped (0); T-shaped, with long, slender lateral processes (1). (Laurin & Reisz, 1995 #92)

(135) Interclavicle attachment for clavicle: ventral sutural area (0); anteriorly directed groove (1); tightly sutured into plastron (2). (Laurin & Reisz, 1995 #93)

(136) Cleithrum: present (0); absent (1). (Modified from Laurin & Reisz, 1995 #94)

(137) If cleithrum is present, it: caps scapula anterodorsally (0); does not cap scapula at all (1). (Modified from Laurin & Reisz, 1995 #94)

(138) Scapula: broad and low (0); narrow and high (1). (Modified from Laurin & Reisz, 1995 #96) When low the height of the scapula is equal to or less than two and a half times its anteroposterior length.

(139) Supraglenoid foramen: present (0); absent (1). (Laurin & Reisz, 1995 #97)

(140) Glenoid: helical, composed of a single facet (0); bipartite, composed of two facets (1). (Modified from Laurin & Reisz, 1995 #98)

(141) Acromion: absent (0); present (1). (Laurin & Reisz, 1995 #99)

(142) Sternum: not mineralized (0); mineralized (1). (Laurin & Reisz, 1995 #100)

(143) Supinator process: strongly angled relative to shaft (0); parallel to shaft (1). (Modified from Laurin & Reisz, 1995 #101)

(144) If supinator process is parallel to shaft it is: separated from it by a groove (0); not separated from shaft (1). (Modified from Laurin & Reisz, 1995 #101)

(145) Ectepicondylar foramen: only groove present (0); groove and foramen present (1); only foramen present (2); both absent (3). (Laurin & Reisz, 1995 #102)

(146) Entepicondylar foramen: present (0); absent or not fully enclosed (1). (Laurin & Reisz, 1995 #103)

(147) Humerus: with robust heads and a short shaft (0); short and robust, without a distinct shaft (1); slender with long shaft (2). A short shaft is one that has a proximodistal length that is equal to or less than the mediolateral width of the heads. (Modified from Laurin & Reisz, 1995 #104)

(148) Olecranon process: present (0); absent (1). (Modified from Laurin & Reisz, 1995 #105)

(149) If present, the olecranon process is: large, with articular facet of ulna facing medially (0); small, with articular facet of ulna facing proximally (1). (Modified from Laurin & Reisz, 1995 #105)

(150) Manual phalangeal formula: 2 3 4 5 3 (0); 2 3 4 4 3 (1); 2 3 3 3 3 or less (2). (Laurin & Reisz 1995, #106)

(151) Dorsolateral shelf on iliac blade: absent (0); present (1). (Laurin & Reisz, 1995 #107)

(152) Iliac blade: low, with long posterodorsal process that extends beyond the posterior edge of the iliac body (0); dorsally expanded, distally flaring, the posterodorsal process does not extended beyond the posterior edge of the iliac body (1). (Laurin & Reisz, 1995 #108)

(153) Acetabular buttress: small, overhangs acetabulum only moderately (0); large, overhangs acetabulum strongly (1). (Laurin & Reisz, 1995 #109)

(154) Oblique ventral ridge of femur (adductor crest): present (0); absent (1). (Laurin & Reisz, 1995 #110)

(155) Femoral proximal articulation: anteroposteriorly long (0); round (1). (Laurin & Reisz, 1995 #111)

(156) Greater trochanter of femur: absent (0); present on posterior edge of femur (1). (Laurin & Reisz, 1995 #112)

(157) Femoral shaft: short and broad (0); long and slender (1). A short and broad femoral shaft is one that has a proximodistal length that is equal to or less than the mediolateral width of the distal head in ventral view (Laurin & Reisz, 1995 #113)

(158) Astragalus: absent (0); present (1). (Modified from Laurin & Reisz, 1995 #115)

(159) If astragalus is present, it: incorporates incompletely fused tibiale, intermedium, and perhaps centrale 4 (0); is without traces of compound origin (1). (Modified from Laurin & Reisz, 1995 #115)

(160) Tibio-astragalar joint: flat (0); tibial ridge fits into astragalar groove (1). (Laurin & Reisz, 1995 #116)

(161) Astragalus and calcaneum: separate (0); sutured or fused (1). (Laurin & Reisz, 1995 #117)

(162) Medial pedal centrale: present (0); absent (1). (Laurin & Reisz, 1995 #118)

(163) Number of distal tarsals: five (0); four or less (1). (Laurin & Reisz, 1995 #119)

(164) Metapodials: not overlapping (0); overlapping (1). (Laurin & Reisz, 1995 #121). When metapodials are not overlapping the carpus or tarsus is short and broad. Likewise, when metapodials are overlapping the carpus or tarsus is long and slender.

(165) Pedal phalangeal formula: 2 3 4 5 4 (0); 2 3 4 4 3 (1); 2 3 3 4 3 or less (2). (Laurin & Reisz, 1995 #122)

(166) Ratio between length of metatarsal I to length of metatarsal IV: at least 0.5 (0); less than 0.5 (1). (Laurin & Reisz, 1995 #123)

(167) Number of maxillary tooth positions: 0-15 (0); 16-30 (1); more than 30 (2). (Modesto et al., 2015)

(168) Number of premaxillary tooth positions: 0-3 (0); 4-6 (1); more than 6 (2). (Modesto et al., 2015)

(169) Single large tooth on anteriormost end of vomer: absent (0); present (1). In some taxa there is a single larger tooth found on the anterior end of the vomer, rather than numerous small teeth. (MacDougall et al., 2017)

(170) Bulbous marginal teeth: absent (0); present (1). Teeth of the maxilla and dentary are considered to be bulbous when the largest teeth have maximum widths that are equal to or more than their maximum height. (MacDougall et al., 2017)

*(171) If present, the lower temporal fenestra morphology is: narrow dorsoventrally (0); tall dorsoventrally (1). (Modified from Benson, 2012 #7) LTF is narrow when temporal fenestra height is less than half the height of the temporal region.

*(172) Postfrontal morphology is: small, occupies approximately one-third of dorsal orbit rim, not transversely broad, and has approximately flat or convex dorsolateral surface (0); dorsolateral surface concave (recessed between orbit and temporal fenestra) (1); long and broad forming prominent supraorbital shelf (2); strongly recessed posterolateral surface forming anterior part of fossa around temporal fenestra (3). (Benson, 2012 #62)

*(173) Jugal, anteroposterior thickness of dorsal ramus (forming postorbital bar): broad, temporal fenestra only weakly emarginates the jugal (0); narrow, jugal strongly emarginated (1). (Benson, 2012 #73)

*(174) If a lower temporal fenestra is present, does the squamosal contribute to the lower temporal bar: no, distinct anteroventral process absent (0); yes (1). (Benson, 2012 #82)

*(175) Snout shape: broad, wider mediolaterally than tall dorsoventrally and nasal primarily on dorsal surface of skull (0); taller than wide, nasal contributes to lateral surface of snout (1).

*(176) Maxilla, lateral surface orientation: vertical or slopes weakly dorsomedially (0); slopes dorsolaterally, overhanging tooth row (1). (Benson, 2012 #21)

*(177) Frontal, anterior process length: short (0); longer than posterior process (1); very long, forming at least 2/3 length of bone (2). (Benson, 2012 #59)

*(178) Posterior dorsal neural spine orientation: approximately vertical (0); posteriormost one or two dorsal neural spines anterodorsally inclined (1); several posterior neural spines anterodorsally inclined (2); strongly posteriorly inclined (3). (Benson, 2012 #165)

REFERENCES

Benson, R. B. J. (2012). Interrelationships of basal synapsids: cranial and postcranial morphological partitions suggest different topologies. *J. Syst. Palaeontol.* 10, 601–624.

deBraga, M., and Reisz, R. R. (1996). The Early Permian reptile *Acleistorhinus pteroticus* and its phylogenetic position. *J. Vertebr. Paleontol.* 16, 384–395.

deBraga, M., and Rieppel, O. (1997). Reptile phylogeny and the interrelationships of turtles. *Zool. J. Linn. Soc.* 120, 281–354.

Laurin, M., and Reisz, R. R. (1995). A reevaluation of early amniote phylogeny. *Zool. J. Linn. Soc.* 113, 165–223.

Lee, M. S. Y. 1993. The origin of the turtle body plan: bridging a famous morphological gap. *Science* 261, 1716–1720.

Lee, M. S. Y. (1995). Historical burden in systematics and the interrelationships of ‘parareptiles’. *Biol. Rev.* 70, 459–547.

Lee, M. S. Y. (1997). Pareiasaur phylogeny and the origin of turtles. *Zool. J. Linn. Soc.* 120, 197­–280.

MacDougall, M. J, and Reisz, R. R. (2014). The first record of a nyctiphruretid parareptile from the Early Permian of North America, with a discussion of parareptilian temporal fenestration. *Zool. J. Linn. Soc.* 172, 616–630.

MacDougall, M. J., Scott, D., Modesto, S. P., Williams, S. A., and Reisz, R. R. (2017). New material of the reptile *Colobomycter pholeter* (Parareptilia: Lanthanosuchoidea) and the diversity of reptiles during the Early Permian (Cisuralian). *Zool. J. Linn. Soc.* 180, 661–671.

Modesto, S. P. (1999). Observations on the structure of the Early Permian reptile *Stereosternum tumidum* Cope. *Palaeontol. Afr.* 35, 7–19.

Modesto, S. P., Scott, D. M., MacDougall, M. J., Sues, H. -D., Evans, D. C., and Reisz, R. R. (2015). The oldest parareptile and the early diversification of reptiles. *Proc. R. Soc. Lond. B Biol. Sci.* 282, 20141912.

Müller, J., and Tsuji, L. (2007). Impedance-matching hearing in Paleozic reptiles: evidence of advanced sensory perception at an early stage of amniote evolution. PLoS ONE 2, e889.

Reisz, R. R., Müller, J., Tsuji, L., and Scott, D. (2007). The cranial osteology of *Belebey vegrandis* (Parareptilia: Bolosauridae), from the Middle Permian of Russia, and its bearing on reptilian evolution. *Zool. J. Linn. Soc.* 151, 191–214.

Tsuji, L. A. (2006). Cranial anatomy and phylogentic affinity of the Permian parareptile *Macroleter poezicus*. *J. Vertebr. Paleontol.* 26, 849–865.
